# Enhancing Agency in Individuals with Depressive Symptoms: The Roles of Effort, Outcome Valence, and Its Underlying Cognitive Mechanisms and Neural Basis

**DOI:** 10.1155/2024/3135532

**Published:** 2024-06-27

**Authors:** Jingyuan Lin, Xuemei Yang, Hong Li, Wuji Lin, Jie Zhang, Yi Lei

**Affiliations:** ^1^The Institute of Brain and Psychological Science, Sichuan Normal University, Chengdu, China; ^2^College Students Mental Health Education Service Center, Sichuan Normal University, Chengdu, China; ^3^College of Psychology, Sichuan Normal University, Chengdu, China; ^4^School of Psychology, South China Normal University, Guangzhou, China

## Abstract

**Background:**

Agency, a sense of control over one's actions and outcomes, is crucial for recovery from depressive symptoms. However, the mechanisms that enhance agency in individuals with depressive symptoms remain poorly understood. This study endeavors to elucidate these fundamental processes.

**Materials and Methods:**

We recruited 52 participants exhibiting depressive symptoms to participate in a novel Judgment of Agency (JoA) task. This task was structured with a 3 (effort: high load, medium load, low load) × 2 (outcome: win, miss) within-subject design to assess the impact of effort and outcome valence on agency. Throughout the task, we utilized functional near-infrared spectroscopy (fNIRS) to explore the neural mechanisms underlying agency. Furthermore, we conducted a randomized, sham-controlled, pre–post-test trial involving intermittent theta-burst stimulation (iTBS) targeted at the left dorsolateral prefrontal cortex (DLPFC) to investigate its potential to enhance agency. Participants were randomly allocated to either an active iTBS group or a sham group, with each receiving a single session of stimulation (600 pulses). The JoA task was conducted both before and after the stimulation.

**Results:**

Effort significantly influenced agency in individuals with depressive symptoms, with this effect being moderated by the outcomes' valences. Agency was positively correlated with self-efficacy (*r* = 0.28, *P* < 0.05) when goals were achieved with effort, and with anxiety severity (*r* = 0.29, *P* < 0.05) when goals were not achieved. Additionally, it was associated with the activation of several frontal brain regions (all *P* values < 0.01), including the left DLPFC, right premotor and supplementary motor areas, and the left inferior frontal gyrus (IFG). Application of iTBS over the left DLPFC significantly enhanced self-attributed agency, particularly when the outcomes were achieved under conditions of low-load effort.

**Conclusions:**

Our study highlights the critical role of effort in enhancing agency for individuals with depressive symptoms, with iTBS applied to the left DLPFC showing potential to enhance agency postgoal achievement. Moreover, the activation of the left IFG and the presence of anxiety are associated with maladaptive self-attributed agency, offering potential targets for therapeutic intervention.

## 1. Introduction

Individuals with depressive symptoms face a significantly increased risk of developing major depressive disorders, which underscores the importance of early interventions to mitigate the prevalence of severe depression and the associated healthcare burdens [1, 2]. Agency, which is defined as a sense of control over one's actions and outcomes [3, 4, 5], is essential for recovery from depression as it promotes autonomy, treatment adherence, and therapeutic progress [6, 7, 8, 9]. However, the factors that enhance agency in individuals with depressive symptoms are not well understood. Considering the critical role of sustained efforts, such as regular exercise, in managing depressive symptoms, and given the variability in treatment responses [10], understanding how effort and outcome valence interact to influence agency is essential. Such insights are crucial for developing targeted interventions to improve the effectiveness of treatments for depressive symptoms.

In the general population, numerous studies have shown that outcomes congruent with predictions or with positive valence can enhance agency [11, 12]. This association may arise from a cognitive tendency that predisposes individuals to attribute positive events or outcomes to their own actions and abilities, known as self-serving biases [13]. Conversely, negative events or outcomes are generally attributed to external factors perceived as beyond one's control. However, individuals with depressive symptoms often exhibit a diminished self-serving bias [14, 15, 16], which may potentially attenuate the impact of outcome valence on their agency. This hypothesis is supported by Mehta et al. [17], who observed that patients with mood disorders, including depression, showed reduced agency following positive outcomes compared to healthy individuals. However, their study did not examine the effects of effort loads on agency.

The relationship between effort on agency is complex, with philosophical and empirical discourses enriching the understanding of this dynamic [18, 19]. Effort has long been a focal point in philosophical deliberations on agency [20, 21]. Empirical research indicates a complex interplay: Planned actions may reduce perceived agency and effort [22], while increased physical and cognitive effort has been shown to have a positive correlation with agency [18, 23]. However, Barlas et al. [19] found no correlation, and in some cases, high-load effort was associated with a decrease in agency [24, 25], suggesting a conditional effect of effort on agency [26, 27]. Specifically, effort enhances the value of goals and satisfaction [28], but also incurs costs, such as fatigue [29, 30, 31]. This suggests that the influence of effort on agency may be moderated by the interaction between effort loads and outcome valences. Notably, depression is associated with reduced willingness to expend effort for rewards, particularly for physical effort expenditure (e.g., rapidly pressing buttons). For individuals with depressive symptoms, the perceived cost of physical effort may be heightened and its value diminished due to symptoms such as psychomotor retardation and avolition [32, 33], increased vulnerability to fatigue [31], and deficits in interest or pleasure in activities [34, 35]. Therefore, it is especially important to consider how effort and outcome valence affect individuals with depressive symptoms.

The Judgment of Agency (JoA) task is a critical instrument for exploring the cognitive foundations of agency. Synofzik et al. [36] proposed that the JoA captures the explicit, conscious cognitive aspect of agency, which is crucial for human self-perception [37]. The task evaluates an individual's control over outcomes by altering action execution and outcome processing, and it uses rating scales or yes/no responses posttrial to measure the sense of agency [38, 39, 40]. Typically, higher JoA scores indicate a stronger sense of self-attributed agency, while lower scores may indicate weaker self-attributed agency or external-attributed agency, where outcomes seem to be determined by external factors. The Optimal Cues Integration Framework provides a theoretical basis for these processes [36, 41, 42]. It suggests that the mind integrates predictive cues, such as action selection and motor output, with postdictive cues like contextual estimations and outcome processing, assigning weights based on reliability to ascertain agency [36]. Leveraging this framework, we designed a novel JoA task that manipulates physical effort and outcome valence to elucidate the cognitive mechanisms by which these factors influence agency in individuals with depressive symptoms.

Neuroimaging techniques have been instrumental in uncovering the neural basis of agency [43, 44, 45, 46, 47, 48]. For instance, Chambon et al. [49] found that reduced activity in the left dorsolateral prefrontal cortex (DLPFC) during action selection was negatively correlated with agency. This decrease in activity was notably associated with the active state of the angular gyrus (AG) within the left inferior parietal cortex (IPC). Additionally, Wakata and Morioka [50] used functional near-infrared spectroscopy (fNIRS) to show that an increase in self-attributed agency, observed while watching congruent hand grasping movements, was linked to higher oxyhemoglobin levels in the right prefrontal region. Neural activity in the frontal lobe is positively associated with agency enhancement, contrasting with the effects seen in the AG. fNIRS offers a cost-effective alternative to fMRI, delivering valuable insights into cortical activity and being suitable for naturalistic settings [51]. As a result, in this study, we employed fNIRS to monitor oxyhemoglobin changes continuously in the agency-relevant cortical areas during the JoA task.

Transcranial magnetic stimulation (TMS) can facilitate or inhibit cortical excitability, thereby elucidating the specific directional effects of cortical activity involved in the processing of agency [49, 52]. Chambon et al. [53] used a JoA task in conjunction with single-pulse TMS to ascertain the role and mechanism by which the IPC, particularly the AG, contributes to agency. Their findings indicated that TMS targeted over the IPC could disrupt the predictive and action execution processes associated with agency in healthy adults. In patients with functional neurological disorder, Bühler et al. [54] applied theta-burst repetitive TMS (iTBS vs. cTBS vs. sham) over the right temporoparietal junction (rTPJ), suggesting that intermittent theta-burst stimulation (iTBS) could enhance cortical excitability related to agency. It is noteworthy that depressive symptoms, which are frequently linked to hypoactivity in the DLPFC during tasks involving cognitive control, emotional regulation, and effort-based decision-making [55, 56, 57], have demonstrated responsiveness to iTBS modulation [58, 59, 60, 61, 62]. Nonetheless, the efficacy of iTBS over the left DLPFC in enhancing agency in this population remains an open question.

Collectively, this study aims to achieve three primary objectives: (1) to elucidate the role of effort and outcome valence on agency in individuals with depressive symptoms and to investigate its underlying cognitive mechanisms, (2) to identify the neural basis associated with these processes, and (3) to examine whether iTBS over the left DLPFC can enhance agency in this population.

## 2. Materials and Methods

### 2.1. Participants

Considering the potential for participant dropouts and in line with previous studies that have recruited between 16 and 25 participants per experiment [17, 18, 19, 48, 63], we recruited 55 college students who scored above the BDI-II threshold for depressive symptoms (score ≥ 14) from an initial pool of 299 respondents. After excluding three participants due to incompatibilities with the fNIRS cap size, our final sample included 52 participants (mean age 19.68 ± 0.96 years).

Upon examining the iTBS effects, participants were randomized into either the iTBS group (*n* = 26, 22 females, mean age 19.54 ± 0.9 years) or the sham group (*n* = 26, 20 females, mean age 19.85 ± 1.01 years). This sample size exceeded the minimum of 21 participants as determined by our a priori power analysis using G^*∗*^Power 3.1.0 [64], ensuring adequate statistical power (detailed calculations are provided in Appendix S1).

All participants were right-handed, with normal or corrected vision, free from organic head disease, metal implants, or a history of epilepsy, and had no prior experience with antidepressants or TMS. Informed consent was obtained voluntarily from each participant, and they received a compensation of 150 RMB upon study completion. The study was approved by the SCNU-20210530 Ethics Committee and was conducted in accordance with the Declaration of Helsinki.

### 2.2. Study Design and Procedure

To elucidate the role of effort and outcome valence on agency in individuals with depressive symptoms, and to investigate its underlying cognitive mechanisms and neural basis, we developed a novel JoA task (details provided in the subsequent section). This task utilized a 3 (effort: high load, medium load, low load) × 2 (valence: win, miss) within-subject design. We applied fNIRS (details in the fNIRS setup) to continuously monitor changes in blood oxygenation during the JoA task. To examine the role of iTBS over the left DLPFC on agency, we employed a randomized, sham-controlled, and pre–post-test design.

**Figure 1:**
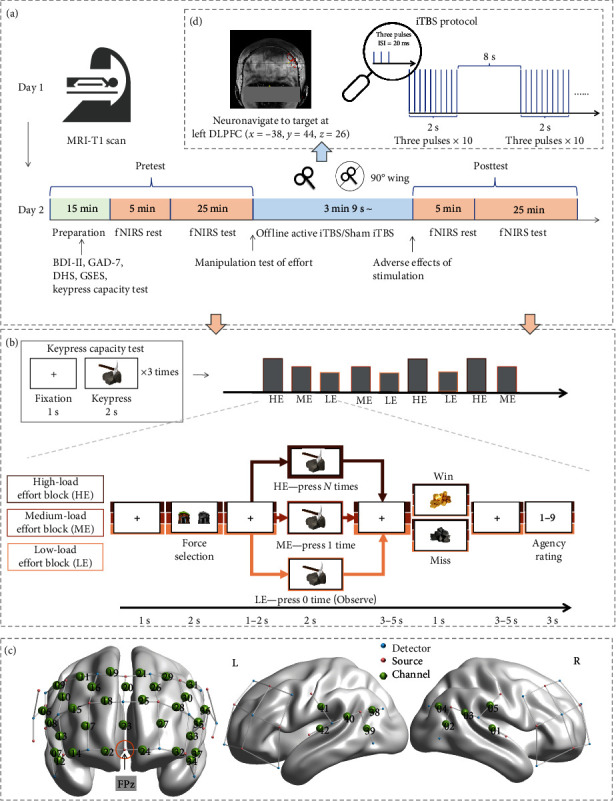
Outline of experimental settings. (a) Flowchart of the experiment. BDI-II = Beck Depression Inventory-II, GAD-7 = Generalized Anxiety Disorder-7, DHS = Dispositional Hope Scale, GSES = General Self-efficacy Scale. (b) Timeline of an example trial of three-load effort blocks in the judgment of agency task (golden miner task). (c) Sources (pink), channels (green with channel numbers), and detectors (blue) were used in the functional near-infrared spectroscopy (fNIRS) recording. (d) Intermittent theta burst stimulation (iTBS) protocol.

Participants visited the laboratory twice (Figure 1(a)). On day 1, they underwent a T1-weighted structural MRI scan using a Siemens 3.0T scanner with the following parameters: TR = 2,530; TE = 3.0; FoV = 224 mm × 256 mm. On day 2, they completed two sessions of the JoA tasks, with an iTBS or sham stimulation session in between. Initially, participants provided demographic information and completed an array of psychometric scales assessing depression (BDI-II) [65], anxiety (Generalized Anxiety Disorder-7) [66], dispositional hope (Dispositional Hope Scale) [67], and self-efficacy (General Self-efficacy Scale) [68]. Then, they engaged in the first JoA task session (pre-iTBS test) to explore the impact of effort and outcome valence on agency. Following this, they evaluated their effort engagement and liking for each condition and rated the perceived task difficulty and win ratio. Following these assessments, participants were randomly assigned to either iTBS active group or sham group and received a stimulation session (detailed protocol in the iTBS section). Subsequently, we inquired about the adverse effects, including pain sensation and level of tolerability, using measures from Blumberger et al. [58]. Finally, they engaged in the second JoA task session (post-iTBS test). For comprehensive details on the scales used, please refer to Appendix S2.

### 2.3. Judgment of Agency Task

In the JoA task depicted in Figure 1(b), we used a cover story to informed participants that they were taking part in a “Golden Miner Game,” with the objective of acquiring as many gold coins as possible. The story with the following instruction in *italics*:

*“At the beginning of each round of the game, there are two holes, one containing gold coins and the other without. The gold coins are buried at certain distance range (“D” meters) beneath the hole, and this range is adjusted based on your keypress capacity (“K”), varying within the range from K-5 to K+5 meters. However, no matter what, you can only obtain the gold coins by choosing the correct holeAND reaching or exceeding the buried distance of the gold through keypress operations. In each round of the task, you will be instructed to choose one of the holds, and then digging by pressing the keys in different ways according to the requirements of different effort conditions. Receiving gold coins signifies that the goal for this round is achieved (win), while receiving stones signifies that the goal is not met (miss). Therefore, for you, you must dig as hard as possible to increase the probability of obtaining gold coins (achieving goals).”*

This task consists of the following four key parts:

Keypress capacity test. Initially, participants were instructed to repeatedly press the spacebar as fast as possible using their left index finger for 2 s once a mining signal manifested after a 1-s “+” fixation. After three attempts, the program computed the average keystrokes per 2 s, termed “*K*,” representing the individual's keypress capacity. Then, they were asked to read the cover story.

Mine hole selection. Post a practice round and confirmation of task comprehension, each trial began with a 1-s “+” fixation, followed by an image displaying two mine holes. They were instructed to choose the colored holes instead of the gray one by pressing “←” or “→” with their right index or middle finger accordingly.

Effort-based digging. After a jitter 1–2 s interval, a 2-s mining signal was displayed. The specific keypress operations for the three levels of effort and the rules for coin acquisition are detailed in Table 1.

Agency judgment. Following a jitter 3–5-s interval, participants were shown a 1-s outcome, which was either gold coins (win) or stones (miss). This was followed by another jitter 3–5-s interval. Participants rated their perceived agency on a 1–9 scale, with 1 indicated “no control (the outcome completely controlled by others),” 9 represented “control (the outcome completely controlled by self),” and the midpoint (5) indicating “uncertain” agency.

The JoA task included three effort conditions: high load, medium load, and low load, with each effort condition encompassing three blocks. Each block presented eight trials with randomized win-or-miss outcomes, and block sequences were pseudo-randomized (further details are provided in Appendix S3). Therefore, the outcomes were no controlled by the participants' actions. To obscure participants' prediction regarding the outcome, we varied the win ratio within each block of identical effort conditions at 37.5%, 50%, and 62.5%.

The experiment was programed using Presentation 0.71 software (Neurobehavioral Systems, Albany, California). Visual stimuli, sized 400 × 311 pixels, were presented on an HP monitor (resolution 2,560 × 1,600, 60-Hz refresh rate) positioned 80 cm in front of the participants.

### 2.4. fNIRS Setup

To identify the neural underpinnings engaged during the JoA task, we utilized a NIRS setup (NirsScan, Huichuang, China). This setup comprised 42 channels, formulated by 16 sources and 17 detectors (Figure 1(c)). We captured continuous waves NIRS signals at an 11-Hz sampling rate, within a wavelength spectrum of 730–850 nm.

The optical probe was positioned using a NIRS-electroencephalogram (EEG)-compatible cap conforming to the international 10–20 electrode placement system (EasyCap, Herrsching, Germany). Source 9 was aligned with the FPz, representing the midline frontal electrode in the EEG system. With a mean separation of approximately 3 cm between the source and detector, each channel's location was determined as the midpoint. We employed the NFRI tool to deduce each channel's Montreal Neurological Institute (MNI) coordinates [69]. The channel-to-brain region mappings were guided by the adult Brodmann Talairach Brain Atlas, focusing on areas pivotal to the agency, particularly the bilateral prefrontal and temporoparietal AG. Furthermore, the mapping included brain regions essential for finger movement, such as the premotor cortex and the supplementary motor areas (pre-SMA), which are recognized for their critical function in the planning and execution of complex motor activities. Detailed mappings are provided in Appendix S4.

### 2.5. iTBS Protocol

To examine whether iTBS over the left DLPFC can enhance agency, we used a figure-eight coil with a PowerMAG stimulator (MAG and More GmbH, Germany) for the administration of TBS. Neuronavigation (Visor2, ANT Neuro GmbH, Germany) guided the precise positioning of the coil over the left dorsolateral prefrontal cortex (DLPFC), targeting the coordinates (*x* = −38, *y* = 44, *z* = 26) (Figure 1(d)).

We determined the resting motor threshold (RMT) by administering single pulses to the left primary motor cortex (M1), with RMT defined as the minimum intensity needed to elicit motor-evoked potentials >0.05 mV in half of 10 trials. Active iTBS was delivered at either 100% (*n* = 19) or adjusted to 80% (*n* = 7) of RMT, based on participant's comfort, using a 50-Hz frequency and a 600-pulse/session protocol. The sham group received stimulation with identical pulse parameters but with the coil angled at 90° from the scalp, employing only one wing in contact with the left DLPFC (the one-wing 90° technique), as detailed by Lisanby et al. [70]. No participants withdrew due to adverse effects from either active iTBS or sham stimulation.

### 2.6. Data Analysis

#### 2.6.1. Behavioral Analysis

First, we tested the discriminability of the effort manipulation. Subsequently, to explore the impact of effort and outcome valence on agency, we performed a 3 (effort: high load, medium load, low load) × 2 (outcome: win, miss) analysis of variance (ANOVA) on the agency ratings. Additionally, we compared the agency ratings to the midpoint (5) using one-sample *t*-tests to understand whether agency was self-attributed, external attributed, or uncertain under each condition. Additionally, we conducted a correlation analysis to explore the relationship between participant characteristics—such as depression, anxiety, hope, and self-efficacy—and agency ratings during the JoA task, which aids in a deeper understanding of the nature of agency under different conditions. Finally, to investigate whether iTBS over the left DLPFC can enhance agency, we applied a 2 (test: pretest, posttest) × 3 (effort: high load, medium load, low load) × 2 (group: iTBS, sham) analysis of covariance, controlling for self-reported pain and intolerance. Agency ratings following win or miss outcomes were analyzed as distinct dependent variables. All behavioral data were processed using JASP 0.17.2.1 (JASP Team, Amsterdam, The Netherlands).

#### 2.6.2. fNIRS Preprocessing

We employed the Homer2 toolkit, which entailed tasks like light intensity detection, transformation to optical density, motion artifact management (tMotion = 0.5; tMask = 1.0; STDEVthresh = 50; AMPthresh = 5.00; *P*=0.99; turnon = 1), band-pass filtering (range: 0.01–0.5 Hz), and conversion of optical density readings to variations in blood oxygenation (*Δ*HbO and *Δ*HbR). Given its sensitivity to cerebral blood flow fluctuations, *Δ*HbO was applied to derive the *β*-value through the general linear model analysis in the NIRS-KIT toolkit [71].

#### 2.6.3. fNIRS Data Analyses

To investigate the neural substrates underlying effort execution and outcome processing during the JoA task, we initiated our analysis at the individual level. *β*-values were ascertained for the three effort conditions (with onset synchronized to the block initiation) and for the six outcome scenarios (resulting from the combination of three effort levels and two outcomes, with onset aligned to the display of the outcome image and a 3-s duration). This process involved convolving the changes in *Δ*HbO with the canonical hemodynamic response function (HRF). Subsequently, at the group levels, we employed a one-sample *t*-test (with a comparison to “0” and a one-tailed approach), using False discovery rate (FDR) correction to highlight active channels associated with each experimental condition. This was followed by a more comprehensive ANOVA that integrated the significant experimental conditions and channels identified in the preceding *t*-tests. Furthermore, network analysis was implemented to explore the interplay between neural activity and agency ratings. To examine the neural basis of the effects of iTBS on agency, we conducted paired sample *t*-tests (with FDR correction) on the pre- and post-test *β*-values for both the active and sham groups to identify brain regions exhibiting iTBS-specific changes.

A threshold of *P* < 0.05 was considered significant. If the sphericity assumption violated, we reported Greenhouse–Geisser spherical corrected degrees of freedom (*df*) and *P*-values, resorting to the Bonferroni approach for post hoc test *P*-value adjustments.

## 3. Results

Table 2 provides an overview of the demographic characteristics of our participants, including their scores on various psychological assessments such as depression, anxiety, hope, and self-efficacy. Additionally, it details task-specific metrics like keypress capacity, perceived difficulty, and win ratio. When comparing the active iTBS group with the sham group, we observed significant intergroup differences solely in the levels of perceived pain and intolerance experienced by the participants.

### 3.1. Manipulation Test of Effort

**Figure 2:**
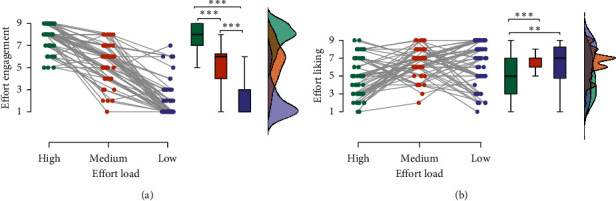
Manipulation test of effort. (a) Self-reported engagement in the three-load effort condition. (b) Self-reported liking of the three-load effort condition. *⁣*^*∗∗*^*P* < 0.01, *⁣*^*∗∗∗*^*P* < 0.001.

We performed a one-way repeated measures ANOVA with three levels of effort (high load, medium load, low load), using self-reported effort engagement as the dependent variable. A significant main effect of effort was observed (*F* (1.92, 97.99) = 225.56, *P*  < 0.001, *ηp^2^* = 0.82), indicating that participants exerted the most effort under the high-load condition (*M* = 7.71, SD = 1.16), followed by the medium-load condition (*M* = 5.42, SD = 1.75), and least under the low-load condition (*M* = 2.08, SD = 1.67) (*P*s < 0.001, Figure 2(a)). A similar analysis was conducted for effort liking, yielding a significant main effect of effort (*F* (1.72, 87.74) = 8.36, *P* < 0.001, *ηp^2^* = 0.14). Participants displayed a lower preference for the high-load condition than the medium-load (*t* = −3.79, *P* < 0.001, Cohen's *d* (*d*) *=* −0.78) and low-load conditions (*t* = −3.22, *P*=0.005, *d =* −0.67), with no significant difference between medium- and low-load conditions (*t* = 0.57, *P* > 0.05, *d =* 0.12) (Figure 2(b)).

### 3.2. Effects of Effort and Outcome on Agency

**Figure 3:**
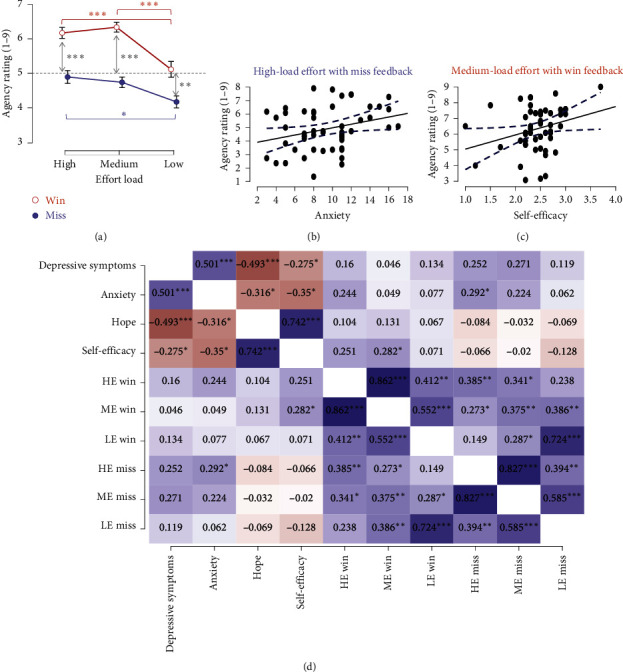
The effect of effort and outcome on judgment of agency and behavioral correlation analysis. (a) The interaction effects of effort loads and outcome valences on agency rating. (b) Scatterplots of the positive correlation between anxiety and agency in the high-load effort with miss outcome condition. The blue dotted line represents the 95% confidence interval. (c) Scatterplots of the positive correlation between self-efficacy and agency in the medium-load effort with win outcome condition. The blue dotted line represents the 95% confidence interval. (d) The Pearson correlation analysis matrix of the participants' characteristics and agency ratings during the pre-iTBS test. HE = high-load effort, ME = medium-load effort, LE = low-load effort. *⁣*^*∗*^*P* < 0.05; *⁣*^*∗∗*^*P* < 0.01; *⁣*^*∗∗∗*^*P* < 0.001.

We conducted a repeated measures ANOVA with a 3 (effort: high load, medium load, low load) × 2 (outcome: win, miss) design, taking the agency rating as the dependent variable. We observed significant main effects of effort (*F* (1.22, 45.20) = 13.43, *P* < 0.001, *ηp*^2^ = 0.21) and outcome (*F* (1,126) = 42.16, *P* < 0.001, *ηp*^2^ = 0.45). Additionally, there was a notable interaction between effort and outcome (*F* (1.46, 3.81) = 5.94, *P*=0.004, *ηp*^2^ = 0.10) (Figure 3(a)). For the win outcome, agency ratings between the high-load and medium-load conditions were not significantly different (*t* = −0.75, *P* > 0.05, *d =* −0.10), yet both were significantly higher than the low-load condition (*P*s < 0.001). For the miss outcome, the high-load condition resulted in significantly higher agency than the low-load condition (*t* = 3.28, *P*=0.02, *d =* 0.43), while the medium-load condition showed no significant difference from the other conditions (*P*s > 0.05). Across all effort conditions, higher agency was associated with the win outcomes than the miss outcomes (*P*s < 0.01), with no other significant observations (*P*s > 0.05).

Using a one-sample *t*-test, we compared agency ratings from each condition to the midpoint “5.” We found that the high load with win outcome (*t* = 6.03, *P* < 0.001, *d =* 0.84) and medium load with win outcome condition (*t* = 6.86, *P* < 0.001, *d =* 0.95) displayed significantly higher agency (indicative of self-attributed agency). Conversely, the low load with miss outcome condition (*t* = −3.25, *P*=0.002, *d = −*0.45) registered significantly reduced agency (pointing to external-attributed agency). All other comparisons remained statistically nonsignificant, suggesting uncertain agency attributions.

### 3.3. Correlation Analysis

Figure 3(b) illustrates a positive correlation between anxiety and agency under conditions of high-load effort with miss outcomes, with a moderate effect size (*r* = 0.29, *P* < 0.05). In a contrasting scenario, Figure 3(c) presents a positive association between self-efficacy and agency under medium-load conditions where outcomes are win (*r* = 0.28, *P* < 0.05). Importantly, we found a strong positive correlation between depression and anxiety (*r* = 0.50, *P* < 0.001). Additionally, depression shows a marked inverse relationship with both hope (*r* = −0.49, *P* < 0.001) and self-efficacy (*r* = −0.28, *P* < 0.05).

### 3.4. Neural Activity of Effort and Outcome Processing

Significant activations (*β*-value > 0, *P*_FDR_ < 0.01) were detected for the high-load effort condition and medium load with the win outcome condition but not in other conditions.

**Figure 4:**
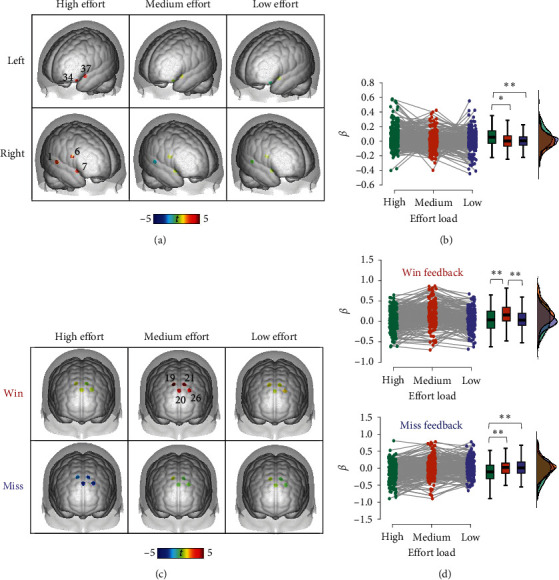
fNIRS results of effort and outcome processing during the judgment of agency task. (a) The observed activated channels in the high-load effort condition. ch1 (*x* = 73, *y* = −23, *z* = 8) corresponds to BA22-superior temporal gyrus (STG); ch6 (*x* = 64, *y* = −13, *z* = 24) corresponds to BA6-pre-motor and supplementary motor area (pre-SMA); ch7 (*x* = 55, *y* = −20, *z* = −9) and ch37 (*x* = −54, *y* = −17, *z* = −9) correspond to BA38-temporopolar area (TP); ch34 (*x* = −54, *y* = 40, *z* = −9) corresponds to BA47-inferior frontal gyrus (IFG). (b) Main effect of effort load on *β*-value (block based). (c) The observed activated channels in the medium-load effort with positive outcome conditions. ch19 (*x* = 12, *y* = 44, *z* = 45), ch20 (*x* = 0, *y* = 53, *z* = 43), ch21 (*x* = −11, *y* = 44, *z* = 54) and ch26 (*x* = −22, *y* = 51, *z* = 43) correspond to BA9-DLPFC. (d) The main effect of effort load on *β*-value in the win and miss outcome condition (trial based). *⁣*^*∗*^*P* < 0.05; *⁣*^*∗∗*^*P* < 0.01.

For high-load effort (Figure 4(a)), five subregions of the temporal gyrus and the frontal cortex exhibited activation: the right superior temporal gyrus (STG, ch1 (*t* = 2.97, *P*=0.005)), right pre-SMA (ch6 (*t* = 3.17, *P*=0.002)), left inferior frontal gyrus (IFG, ch34 (*t* = 3.28, *P*=0.002)), and bilateral temporopolar (TP, ch7 (*t* = 3.53, *P*  < 0.001); ch37 (*t* = 3.59, *P* < 0.001)). Using a 3 (effort: high load, medium load, low load) × 5 (channel: ch1, ch6, ch7, ch34, ch37) ANOVA, with the *β*-value as the dependent variable, there was a significant main effect of effort (*F* (1.91, 97.44) = 6.15, *P*=0.004, *ηp*^2^ = 0.11). However, neither the main effect of the channel nor their interaction was significant (*P*s > 0.05). Post hoc analyses indicated the *β*-value for the high-load condition surpassed those of the medium-load (*t* = 2.97, *P*=0.011, *d* = 0.46) and the low-load conditions (*t* = 3.10, *P*=0.008, *d* = 0.48) (Figure 4(b)).

For medium-load effort with win outcome, activations centered on the DLPFC, as shown in Figure 4(c) (ch19, ch20, ch21, ch26 (*P*s < 0.001)). We applied a 3 (effort: high-load, medium-load, low-load) × 2 (outcome: win, miss) × 4 (channel: ch19, ch20, ch21, ch26) ANOVA, using the *β*-value as the dependent variable. The findings showed significant main effect of effort (*F* (2,102) = 15.09, *P* < 0.001, *ηp*^2^ = 0.23), outcome (*F* (1,51) = 27.84, *P* < 0.001, *ηp*^2^ = 0.35), and their interaction (*F* (2,102) = 4.19, *P*=0.018, *ηp*^2^ = 0.08). Post hoc test showed that for win outcomes, the medium-load *β*-value significantly exceeded those of high-load (*t* = 3.94, *P*=0.002, *d* = 0.49) and low-load (*t* = 3.82, *P*=0.003, *d* = 0.48), as depicted in Figure 4(d) (upper panel). Conversely, for the miss outcome (Figure 4(d), lower panel), both the medium-load (*t* = 3.93, *P*=0.002, *d* = 0.49) and low-load condition (*t* = 3.61, *P*=0.006, *d* = 0.45) had *β*-values surpassing the high-load condition. We also noted outcome differences, with higher *β*-value for wins versus misses in high-load (*t* = 4.36, *P* < 0.001, *d* = 0.55) in the medium-load conditions (*t* = 4.38, *P* < 0.001, *d* = 0.56); however, this pattern was absent in the low-effort condition (*t* = 0.93, *P* > 0.05, *d* = 0.12). All other results were non-significant (*P*s > 0.05).

**Figure 5:**
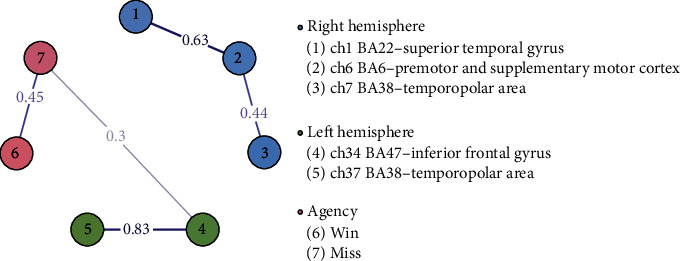
Network plot based on partial correlation estimator. Network plot of the activated channels and agency ratings in the high-load effort condition. Only significant correlation edges (*P* < 0.05) are displayed.

An exploratory network analysis was conducted utilizing the partial correlation estimator (significant set at *P* < 0.05) to assess the functional connectivity between the activated channels and the agency ratings after win and miss outcomes. There was a significant positive correlation between the left IFG and agency after high-load effort with miss outcome (*r* = 0.30) (Figure 5). There was a notable positive correlation between IFG and TP in the left hemisphere (*r* = 0.83). The pre-SMA showed significant positive associations with the STG (*r* = 0.63) and TP (*r* = 0.44) in the right hemisphere, though there was no direct connection with agency ratings (details on centrality measures for each node are available in Appendix S5).

### 3.5. iTBS over the Left DLPFC on Agency

**Figure 6:**
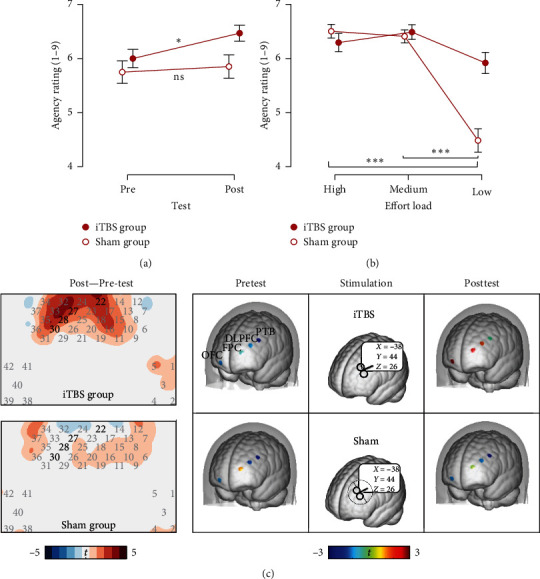
Behavioral and fNIRS results of iTBS over the left DLPFC on the judgment of agency rating in the win outcome condition. (a) The main effect of pre- and post-test on agency. (b) The main effect of effort loads on agency. (c) The activated channels were observed by comparing the posttest and pretest in the iTBS and sham groups. ch22 (*x* = 16, *y* = 72, *z* = 18) corresponds to BA11-orbitofrontal cortex (OFC); ch27 (*x* = −27, *y* = 67, *z* = 14) corresponds to BA10-frontopolar cortex (FPC); ch28 (*x* = −39, *y* = 53, *z* = 27) corresponds to left BA46-DLPFC; ch30 (*x* = −46, *y* = 35, *z* = 35) corresponds to BA45-pars triangularis Broca's area (PTB). *⁣*^*∗*^*P* < 0.05, *⁣*^*∗∗∗*^*P* < 0.001.

To assess the effects of iTBS over the left DLPFC on agency, we segregated the analysis based on win and miss outcomes. For the win outcome, an analysis of covariance was performed with a 2 (test: pretest, posttest) × 3 (effort: high load, medium load, low load) × 2 (group: iTBS, sham) design, taking agency ratings as the dependent variable. There was a significant main effect of test (*F* (1,7.26) = 6.08, *P*=0.017, *ηp*^2^ = 0.11), along with a test × group interaction (*F* (1, 6.48) = 5.43, *P*=0.024, *ηp^2^* = 0.10). The iTBS group displayed a significant rise in agency post-iTBS compared to pre-iTBS (*t* = 3.18, *P*=0.015, *d* = 0.46). In contrast, the sham group showed no significant alteration in agency from the pre- to the post-iTBS test (*t* = 0.82, *P*  > 0.05, *d* = 0.12) (Figure 6(a)). There was also a significant main effect of effort (*F* (1.13,19.59) = 5.79, *P*=0.016, *ηp^2^* = 0.11) and an effort × group interaction (*F* (1.13, 16.37) = 4.83, *P*=0.028, *ηp^2^* = 0.09). For the sham group, while no significant difference was seen between medium-load and high-load effort conditions (*t =* 0.84, *P*  > 0.05, *d =* 0.19), both levels exhibited more agency than the low-load condition (*P*s < 0.001) (Figure 6(b)). The iTBS group revealed no significant differences across efforts (*P*s > 0.05). For miss outcomes, no significant results were detected (*P*s > 0.05).

We performed paired sample *t*-tests between post-iTBS and pre-iTBS test for each group to explore the neural basis of iTBS's effect on agency via the left DLPFC. In the iTBS group, there were significant escalations in *β*-value during the low load with win outcome condition across four channels corresponding to four cerebral regions: orbitofrontal cortex (OFC, ch22 (*t* = 3.68, *P*=0.001)), frontopolar cortex (FPC, ch27 (*t* = 3.50, *P*=0.002)), left DLPFC (ch28 (*t* = 3.55, *P*=0.002)), and left pars triangularis Broca's area (PTB, ch30 (*t* = 3.12, *P*=0.005)). The sham group did not present significant changes in *β*-value (Figure 6(c)).

## 4. Discussion

Enhancing agency in individuals with depressive symptoms is essential for fostering their recovery. This study examined the factors to enhance agency in these individuals and explored the cognitive and neural substrates. Although individuals with depression may typically dislike high-load effort scenarios, our findings indicate that engaging in such efforts significantly enhances their agency—a phenomenon also observed in nondepressed populations [18, 23]. Given the influence of effort on agency, contingent upon win outcome (the achievement of outcome goals), our discussion commences with an exploration of how these goal-achieved efforts positively enhance agency.

### 4.1. Goal-Achieved Effort Enhances Agency in Individuals with Depressive Symptoms

The behavioral data suggest that high-load and medium-load efforts can enhance agency upon achieving goals, as evidenced by higher agency ratings in these conditions than in the low-load effort condition. These conditions displayed agency ratings significantly above the midpoint on the agency rating scale, hinting at a form of self-attributed agency. Interestingly, the mechanisms driving agency within these two goal-achieved effort conditions appear distinct.

High-load effort engagement, despite being an unfavored activity for individuals with depressive symptoms, can enhance their agency. Our manipulation test of effort liking and agency ratings supported this finding. Activation in the subregions of the right temporal lobe (STG, TP), left frontal (IFG), and bilateral temporal lobe (TP) were identified. Of note was activating the right pre-SMA, contralateral to the high-load effort manipulation using the left index finger. Considering these regions' roles in planning and executing goal-directed actions, modulating spontaneous movements, and engendering action awareness [72, 73], this finding suggests a top-down internal control sensation invoked by rapid key pressing. We posit that high-load effort provides subtle internal sensorimotor cues contributing to agency.

We observed a positive relationship between agency and self-efficacy for medium-load effort when participants achieved win outcomes. This finding indicates that achieving goals with moderate effort can enhance agency in those with depressive symptoms, especially if they possess strong self-efficacy. The concept of self-efficacy, a cornerstone of human agency according to Bandura [74], refers to the belief in one's ability to complete a task or surmount a challenge [75]. As higher self-efficacy has been associated with augmented effort toward goal achievement [76], we posit that the salutary impact of goal-achieved medium-load effort on agency in individuals with depressive symptoms may be contingent upon self-efficacy. Concurrently, during the engagement in a medium-load effort that led to positive outcomes, we detected DLPFC activation during the outcome processing, which was associated with self-attributed agency. Given the DLPFC's involvement in volitional actions [46, 47, 49, 77], and in encoding reward-related information [78], we suggest that in our JoA task, moderate effort possibly engaged the DLPFC to merge cues of action autonomy with positive outcomes, leading to a more robust self-attributed agency.

### 4.2. Goal-Unachieved Effort Enhances Maladaptive Agency in Individuals with Depressive Symptoms

In instances where goals were unachieved, the agency under both high-load and medium-load efforts declined, aligning closely with the midpoint on the agency rating scales, signifying ambiguous agency attribution.

The agency rating under the high-load effort condition exhibited a positive correlation with heightened anxiety severity, underscoring a paradoxical “reversal” self-serving bias—an amplified self-attribution of blame during failures—especially in individuals with concurrent depression and anxiety. While a diminished propensity for self-blame can serve as a protective factor that fosters positive self-perceptions [79], the heightened self-attribution observed in anxious individuals when faced with nonachievement may indicate a maladaptive coping mechanism. According to the learned helplessness theory, individuals confronted with overwhelming stressors frequently develop a depressive attribution style [80, 81]. Consequently, individuals with depression and anxiety may be more likely to internalize blame for adverse outcomes, intensifying feelings of worthlessness and diminished motivation [79, 82, 83]. This combination might lead to a propensity to adopt a self-blaming perspective in the face of negative results, reinforcing self-attributed agency even after undesirable outcomes.

Our exploratory network analysis identified a relationship between agency in the goal-unachieved high-load effort condition and the activation of the left IFG. Recognized for its involvement in emotional information processing [84], functional association of the left IFG with the dorsal medial prefrontal cortex/hippocampus can inform the success of subsequent emotional memory retrieval [85]. Within the domain of self-concept processing, a meta-analysis of 59 studies reported that around 55.9% of these investigations implicated the left IFG in self-referential activities, possibly due to its connection to introspective inner speech [86]. We postulate that the heightened agency observed following unmet outcomes might be anchored in the activation of the left IFG. This activation could facilitate the introspective retrieval of internal sensorimotor cues linked to the high-load effort exerted, reducing the propensity to ascribe outcomes to external factors. Notably, a meta-analysis by Marwood et al. [87] exploring neural responsiveness to psychotherapy in depression and anxiety demonstrated that symptom relief correlated with diminished bilateral IFG activation. Drawing on these insights, future research should explore whether suppressing left IFG activity can decrease the maladaptive self-attributed agency in individuals with depression or anxiety.

### 4.3. The Role of iTBS over the Left DLPFC in Enhancing Agency

The application of iTBS to modulate the left DLPFC significantly enhanced agency in the posttest for the iTBS group compared to their pretest ratings. This contrast was absent in the sham group, emphasizing the causal role of the left DLPFC in enhancing agency. This enhancement in agency was observed in goal-achieved conditions. The neuroimaging results corroborated the behavioral findings: post-iTBS modulation, the iTBS group showed increased activation in the left DLPFC, OFC, FPC, and PTB during the low-load effort condition with win outcome. The sham group, on the other hand, displayed negligible neural activity changes between pre- and post-tests. These observations underscore that stimulating the left DLPFC via iTBS can enhance agency during goal achievement. This boost is potentially facilitated by reactivating regions of the middle and left frontal cortex, previously found dormant in depressed individuals during tasks involving reward processing and self-reflective reasoning [88, 89]. Therefore, it is conceivable that the frontal cortex underpins self-reflective and causal reasoning processes central to self-attributed agency, especially in situations with muted sensorimotor cues, such as lower effort conditions.

### 4.4. Theoretical and Clinical Implications

Our study proposes two refinements to the optimal cue integration framework [36, 77]. Based on our results, we recommend integrating “effort” as a pivotal element within sensorimotor prospective cues due to its significance in guiding intentional movements and facilitating self-reflection.

Furthermore, this influence may depend on the severity of anxiety and self-efficacy in individuals with depressive symptoms. There is a pressing need to formulate distinct optimal cue frameworks for agency in the context of positive and negative outcomes, respectively. Emphasizing agency enhancement without considering the variances in outcome might inadvertently produce undesired repercussions. The current theoretical framework fails to explain this situation adequately.

Our empirical findings offer significant implications for the therapeutic landscape of depressive symptoms. Our results emphasize the potential benefits of therapies that involve effort in mitigating depressive symptoms. Individuals with depressive symptoms might shy away from tasks demanding effort, given the fatigue often associated with the condition. However, our evidence suggests that when such effort culminates in positive outcomes, it can significantly enhance self-attributed agency. This increased sense of control may, in turn, foster positive self-perceptions and beliefs, which are paramount in counteracting the negative self-views pervasive in depressive symptoms.

Understanding the intricate relationship between effort, positive outcomes, and agency is crucial for the effective design of interventions. For instance, interventions might begin with activities requiring moderate effort, ensuring early goal achieved, and gradually escalating in complexity and demand. Such a graded approach can enhance self-efficacy—a belief in one's capabilities to accomplish tasks—and serve as a buffer against potential setbacks. Addressing anxiety concurrently is equally critical, as heightened anxiety can be counterproductive, potentially overshadowing the benefits derived from effort.

From a neuroscientific standpoint, our research offers intriguing insights into the neural underpinnings of agency in individuals with depressive symptoms. The prominent role of the left IFG, as revealed in our study, underscores its potential as a therapeutic target. This region's involvement in emotional processing and self-referential activities makes it a linchpin in the neural circuitry of self-attributed agency. Notably, its potential role in internalizing blame upon encountering failures provides a rationale for interventions to modulate its activity. Considering this, inhibitory neuromodulation of the left IFG emerges as a promising avenue. If successful, such interventions could attenuate the propensity of depressed individuals to disproportionately blame themselves for failures, thereby fostering a more balanced and resilient attribution style.

### 4.5. Limitations and Future Directions

Despite the significant findings of this research, there are several limitations.

First, agency measurement. We employed the JoA task to measure agency, which relies on subjective self-reports and targets the explicit and cognitive aspects of agency. However, it may not encompass the implicit dimensions of agency. Further research could integrate both implicit and explicit paradigms, such as the intentional binding [63, 90], to provide a more comprehensive understanding of agency processing in individuals with depressive symptoms.

Second, lack of evaluation by psychiatrists is the weakness of the study. Depressive symptoms were assessed using the BDI-II without formal psychiatric diagnoses. This limitation may mask the complexity of depressive symptomatology, including severity, duration, and comorbidities [91]. Future research should include psychiatric diagnoses to categorize participants based on the severity of their depressive conditions, the chronicity of their symptoms, and the presence of any comorbid disorders. Such a refined approach would allow for a more granular analysis of the relationship between depression and agency, leading to more precise clinical intervention strategies.

Third, sham methodology. This study used a 1-wing 90° sham method as a control for active iTBS. Although this method induces lower brain voltage [70], it also faces several methodological challenges. For instance, the sham operation reduced the somatosensory perception of pain. An ideal sham should mimic sensory effects without direct neural effect, possibly using surface electrodes and shielded TMS coils suggested by Duecker and Sack [92]. Additionally, the adverse effect assessment in this study was limited to pain sensation, potentially overlooking other iTBS-induced effects. Future research should assess a broader range of adverse effects and employ more refined control protocols to confirm the specificity of cortical activity's influence on agency in individuals with depressive symptoms.

## 5. Conclusions

In conclusion, our study has shed light on the critical role of effort in enhancing agency in individuals with depressive symptoms, with the effect being significantly modulated by the interplay of effort loads and the valence of outcomes. Notably, the DLPFC emerges as a key neural region modulating agency, with iTBS over the left DLPFC showing promise in enhancing agency under goal-achieved condition. Additionally, the study illuminates the influence of the left IFG and anxiety on the maladaptive self-attribution of agency under goal-unachieved condition, indicating its potential as a target for therapeutic intervention. These insights pave the way for the development of more precise and efficient intervention strategies aimed at fostering a more adaptive agency, which is vital for the recovery process of individuals with depressive symptoms.

## Figures and Tables

**Table 1:** The keypress operation under different effort conditions and the rules for coin acquisition (achieving goals) in cover story.

Effort condition	Instructions of keypress during mining signal appears	How can I get gold coins
High-load block	Press the spacebar quickly and continuously with your left index finger until the mining signal disappears	The selected hole was correct AND the number of presses ≥ D

Medium-load block	Press the spacebar with your left index finger as fast as possible and hold down the button until the mining signal disappears. The system will calculate the number of presses you make within 2 s based on your reaction time, and this will be considered as the number of presses for this round	The selected hole was correct AND the number of presses that converted from holding time (quicker response times (RTs) or longer holding times, more significant possibility for getting gold coins) ≥ D

Low-load block	Wait for the program to randomly select one of your previous trial performances as the number of presses for this round	The selected hole was correct AND the previous press/hold performance ≥ D

*Note*: D denotes the range of distance underground where gold coins are located. If participants began with the low-load block, their performance would align with the practice phases. Every participant was briefed on this rule prior to the practice.

**Table 2:** Participants' demographic and characteristics.

Variable	Overall, *n* = 52	Group		*χ²/t*	*P*
		iTBS, *n* = 26	Sham, *n* = 26		
Gender	—	—	—	0.5	0.48
Female	42	22	20	—	—
Male	10	4	6	—	—
Age (year)	19.68 (0.96)	19.54 (0.90)	19.85 (1.01)	−1.16	0.25
Depression (BDI-II)	28.37 (7.48)	27.19 (8.41)	29.54 (6.37)	−1.13	0.26
Anxiety (GAD-7)	9.35 (3.5)	9.04 (3.83)	9.65 (3.17)	−0.63	0.53
Dispositional hope (DHS)	40.77 (7.9)	40.27 (8.87)	41.27 (6.94)	−0.45	0.65
Self-efficacy (GSES)	2.43 (0.44)	2.38 (0.48)	2.48 (0.39)	−0.82	0.42
Keypress capacity (K)	13.5 (2.03)	13.00 (1.65)	14.00 (2.28)	−1.81	0.08
Perceptive difficulty	60.39 (18.41)	56.92 (17.89)	63.85 (18.62)	−1.37	0.18
Perceptive win ratio	59 (11.32)	59 (12.62)	59 (10.11)	<0.01	1
Perceived pain	3.15 (2.38)	5.04 (1.93)	1.27 (0.67)	9.42	<0.001
Intolerability of stimulation	3.23 (2.21)	4.23 (2.03)	2.23 (1.95)	3.63	<0.001

## Data Availability

The data supporting the findings are available from the corresponding author upon reasonable request.

## Supplementary Materials

Supplementary Materials Appendix S1: sample size calculation. Appendix S2: manipulation test of effort conditions. Appendix S3: the pseudo-random order matrix of presented nine blocks. Appendix S4: the Montreal Neurological Institute coordinates and the mapping Brodmann area with percentage of overlap of each channel. Appendix S5: centrality measures per node in the high-load effort condition.
