# Supplementary material for: Enhancing Agency in Individuals with Depressive Symptoms: The Roles of Effort, Outcome Valence, and Its Underlying Cognitive Mechanisms and Neural Basis
Source: Depress Anxiety. 2024 Jun 27;2024:3135532. doi: 10.1155/2024/3135532 (PMC11919012; doi:10.1155/2024/3135532)

# Enhancing agency in individuals with depressive symptoms: the roles of effort, outcome valence, and its underlying cognitive mechanisms and neural basis

Supplementary files

Appendix 1 page 2-3

Appendix 2 page 4

Appendix 3 page 5-6

Appendix 4 page 7-8

Appendix 5 page 9

**Appendix 1 Sample size calculation**

To ascertain the appropriate sample size, we performed an a priori power analysis using GPower 3.1.0 (see Figure 1), which is designed to assist in the planning of experiments by calculating the necessary sample size based on a set of defined statistical parameters. Our analysis was based on the following assumptions: an effect size (Cohen’s d) of 0.66, which is recognized as a medium to large effect in the social sciences (Cohen, 1988); an alpha level (α) of 0.05, representing a 5% risk of a Type I error; and a desired statistical power of 0.8, which corresponds to an 80% probability of detecting an effect if it exists (Cohen, 1988). Based on these parameters, GPower indicated that we would require a minimum of 21 participants to adequately power our study to detect the proposed effects.


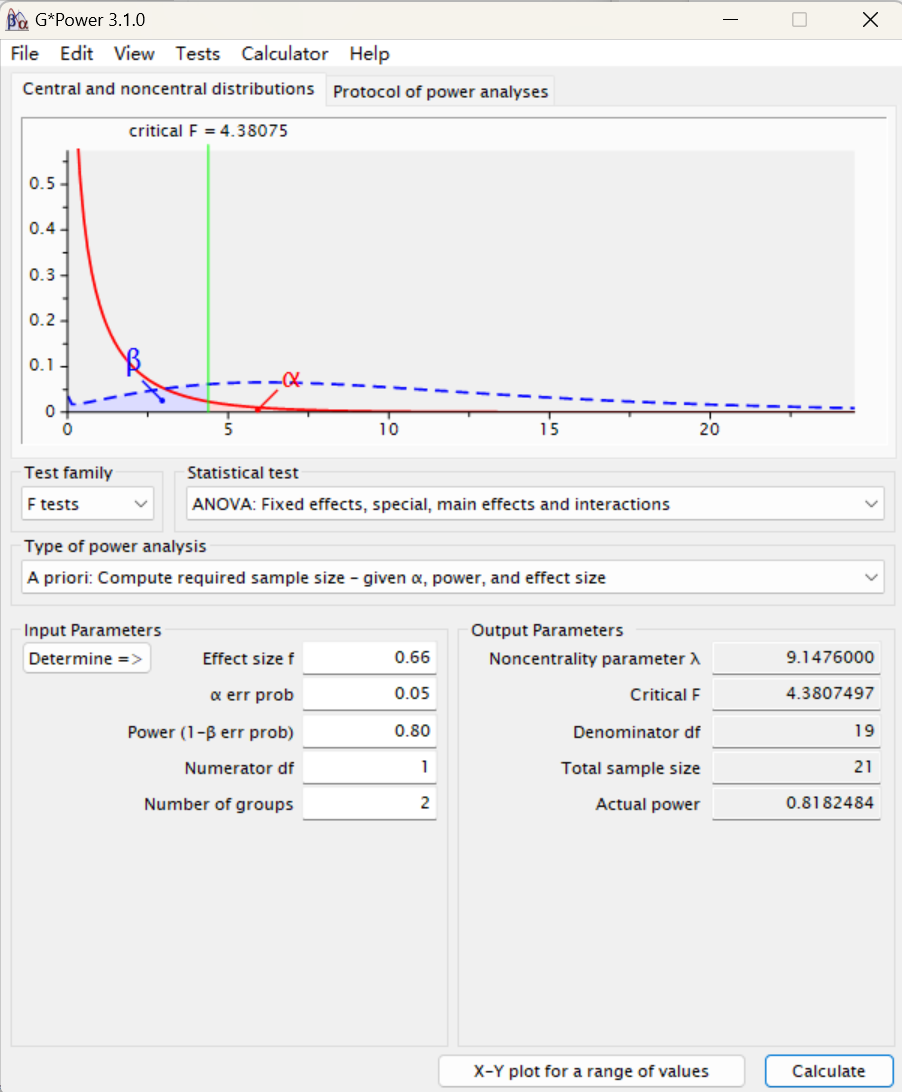


Figure 1 sample size calculation

Additionally, considering the interaction effect between effort and outcome valence in our 3×2 within-subjects design, with a partial eta squared (ηp²) of 0.1, we conducted a post-hoc sample size estimation using MorePower 6.0.4. This analysis (as depicted in Figure 2) revealed that a minimum of 46 participants would be necessary to achieve adequate statistical power. This ensures that our study is adequately powered and not compromised by a lack of sufficient sample size.


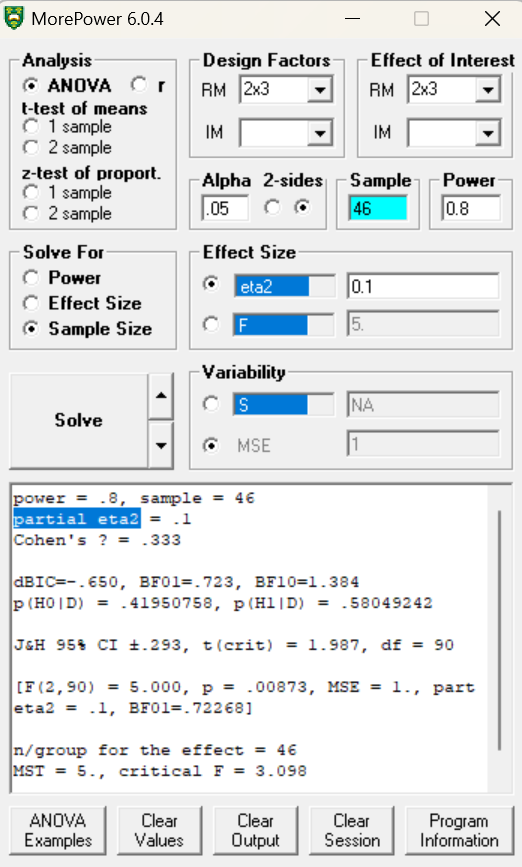


Figure 2 post-hoc sample size calculation

**Appendix 2 Manipulation test of effort conditions**

*Effort engagement on effort conditions*

Q1: How much effort you engaged in the condition of observation (low-load effort)?

1 = not at all; 5 = unclear; 9 = so much

Q2: To what extent you prefer the condition of pressing one times (medium-load effort)?

1 = not at all; 5 = unclear; 9 = so much

Q3: To what extent you prefer the condition of pressing N time (high-load effort)?

1 = not at all; 5 = unclear; 9 = so much

*Liking of effort conditions*

Q1: To what extent you prefer the condition of observation (low-load effort)?

1 = absolutely dislike; 5 = unclear; 9 = absolutely like

Q2: To what extent you prefer the condition of pressing one times (medium-load effort)?

1 = absolutely dislike; 5 = unclear; 9 = absolutely like

Q3: To what extent you prefer the condition of pressing N time (high-load effort)?

1 = absolutely dislike; 5 = unclear; 9 = absolutely like

*Perceptive difficulty of effort condition*

Q1: To what extent you feel difficult about the condition of observation (low-load effort)?

0 = No difficulty at all to 100 = Extremely difficult

Q2: To what extent you feel difficult the condition of pressing one times (medium-load effort)?

0 = No difficulty at all to 100 = Extremely difficult

Q3: To what extent you feel difficult the condition of pressing N time (high-load effort)?

0 = No difficulty at all to 100 = Extremely difficult

*Perceptive win ratio in the Judgment of agency task*

Q1: In the task you just completed, how likely did you feel that you would obtain gold coins?

0% = not win at all to 100% = always win

*Adverse effects of stimulation*

Q1: To what extent you feel pain about the stimulation?

1 = Not at all to 10 = Extremely pain

Q2: To what extent you feel intolerable about the stimulation?

1 = Not at all to 10 = Extremely intolerable

**Appendix 3 The pseudo-random order matrix of presented nine blocks**

| **No.** | **group** | **Pre-test block** | | | | | | | | | **Post-test block** | | | | | | | | |
| --- | --- | --- | --- | --- | --- | --- | --- | --- | --- | --- | --- | --- | --- | --- | --- | --- | --- | --- | --- |
| sub01 | active | 1 | 4 | 9 | 3 | 5 | 8 | 2 | 6 | 7 | 3 | 5 | 8 | 1 | 4 | 9 | 2 | 6 | 7 |
| sub02 | sham | 1 | 4 | 9 | 3 | 5 | 8 | 2 | 6 | 7 | 3 | 5 | 8 | 1 | 4 | 9 | 2 | 6 | 7 |
| sub03 | active | 4 | 9 | 3 | 5 | 8 | 2 | 6 | 7 | 1 | 5 | 8 | 1 | 4 | 9 | 2 | 6 | 7 | 3 |
| sub04 | sham | 4 | 9 | 3 | 5 | 8 | 2 | 6 | 7 | 1 | 5 | 8 | 1 | 4 | 9 | 2 | 6 | 7 | 3 |
| sub05 | active | 9 | 3 | 5 | 8 | 2 | 6 | 7 | 1 | 4 | 8 | 1 | 4 | 9 | 2 | 6 | 7 | 3 | 5 |
| sub06 | sham | 9 | 3 | 5 | 8 | 2 | 6 | 7 | 1 | 4 | 8 | 1 | 4 | 9 | 2 | 6 | 7 | 3 | 5 |
| sub07 | active | 3 | 5 | 8 | 2 | 6 | 7 | 1 | 4 | 9 | 1 | 4 | 9 | 2 | 6 | 7 | 3 | 5 | 8 |
| sub08 | sham | 3 | 5 | 8 | 2 | 6 | 7 | 1 | 4 | 9 | 1 | 4 | 9 | 2 | 6 | 7 | 3 | 5 | 8 |
| sub09 | active | 5 | 8 | 2 | 6 | 7 | 1 | 4 | 9 | 3 | 4 | 9 | 2 | 6 | 7 | 3 | 5 | 8 | 1 |
| sub10 | sham | 5 | 8 | 2 | 6 | 7 | 1 | 4 | 9 | 3 | 4 | 9 | 2 | 6 | 7 | 3 | 5 | 8 | 1 |
| sub11 | active | 8 | 2 | 6 | 7 | 1 | 4 | 9 | 3 | 5 | 9 | 2 | 6 | 7 | 3 | 5 | 8 | 1 | 4 |
| sub12 | sham | 8 | 2 | 6 | 7 | 1 | 4 | 9 | 3 | 5 | 9 | 2 | 6 | 7 | 3 | 5 | 8 | 1 | 4 |
| sub13 | active | 2 | 6 | 7 | 1 | 4 | 9 | 3 | 5 | 8 | 2 | 6 | 7 | 3 | 5 | 8 | 1 | 4 | 9 |
| sub14 | sham | 2 | 6 | 7 | 1 | 4 | 9 | 3 | 5 | 8 | 2 | 6 | 7 | 3 | 5 | 8 | 1 | 4 | 9 |
| sub15 | active | 6 | 7 | 1 | 4 | 9 | 3 | 5 | 8 | 2 | 6 | 7 | 3 | 5 | 8 | 1 | 4 | 9 | 2 |
| sub17 | active | 7 | 1 | 4 | 9 | 3 | 5 | 8 | 2 | 6 | 7 | 3 | 5 | 8 | 1 | 4 | 9 | 2 | 6 |
| sub18 | sham | 7 | 1 | 4 | 9 | 3 | 5 | 8 | 2 | 6 | 7 | 3 | 5 | 8 | 1 | 4 | 9 | 2 | 6 |
| sub19 | active | 7 | 6 | 2 | 8 | 5 | 3 | 9 | 4 | 1 | 8 | 5 | 3 | 7 | 6 | 2 | 9 | 4 | 1 |
| sub20 | sham | 7 | 6 | 2 | 8 | 5 | 3 | 9 | 4 | 1 | 8 | 5 | 3 | 7 | 6 | 2 | 9 | 4 | 1 |
| sub21 | active | 6 | 2 | 8 | 5 | 3 | 9 | 4 | 1 | 7 | 5 | 3 | 7 | 6 | 2 | 9 | 4 | 1 | 8 |
| sub22 | sham | 6 | 2 | 8 | 5 | 3 | 9 | 4 | 1 | 7 | 5 | 3 | 7 | 6 | 2 | 9 | 4 | 1 | 8 |
| sub23 | active | 2 | 8 | 5 | 3 | 9 | 4 | 1 | 7 | 6 | 3 | 7 | 6 | 2 | 9 | 4 | 1 | 8 | 5 |
| sub24 | sham | 2 | 8 | 5 | 3 | 9 | 4 | 1 | 7 | 6 | 3 | 7 | 6 | 2 | 9 | 4 | 1 | 8 | 5 |
| sub25 | active | 8 | 5 | 3 | 9 | 4 | 1 | 7 | 6 | 2 | 7 | 6 | 2 | 9 | 4 | 1 | 8 | 5 | 3 |
| sub26 | sham | 8 | 5 | 3 | 9 | 4 | 1 | 7 | 6 | 2 | 7 | 6 | 2 | 9 | 4 | 1 | 8 | 5 | 3 |
| sub27 | active | 5 | 3 | 9 | 4 | 1 | 7 | 6 | 2 | 8 | 6 | 2 | 9 | 4 | 1 | 8 | 5 | 3 | 7 |
| sub28 | sham | 5 | 3 | 9 | 4 | 1 | 7 | 6 | 2 | 8 | 6 | 2 | 9 | 4 | 1 | 8 | 5 | 3 | 7 |
| sub30 | sham | 3 | 9 | 4 | 1 | 7 | 6 | 2 | 8 | 5 | 2 | 9 | 4 | 1 | 8 | 5 | 3 | 7 | 6 |
| sub31 | active | 9 | 4 | 1 | 7 | 6 | 2 | 8 | 5 | 3 | 9 | 4 | 1 | 8 | 5 | 3 | 7 | 6 | 2 |
| sub32 | sham | 9 | 4 | 1 | 7 | 6 | 2 | 8 | 5 | 3 | 9 | 4 | 1 | 8 | 5 | 3 | 7 | 6 | 2 |
| sub33 | active | 4 | 1 | 7 | 6 | 2 | 8 | 5 | 3 | 9 | 4 | 1 | 8 | 5 | 3 | 7 | 6 | 2 | 9 |
| sub34 | sham | 4 | 1 | 7 | 6 | 2 | 8 | 5 | 3 | 9 | 4 | 1 | 8 | 5 | 3 | 7 | 6 | 2 | 9 |
| sub35 | active | 1 | 7 | 6 | 2 | 8 | 5 | 3 | 9 | 4 | 1 | 8 | 5 | 3 | 7 | 6 | 2 | 9 | 4 |
| sub36 | sham | 1 | 7 | 6 | 2 | 8 | 5 | 3 | 9 | 4 | 1 | 8 | 5 | 3 | 7 | 6 | 2 | 9 | 4 |
| sub37 | active | 1 | 4 | 9 | 3 | 5 | 8 | 2 | 6 | 7 | 3 | 5 | 8 | 1 | 4 | 9 | 2 | 6 | 7 |
| sub38 | sham | 1 | 4 | 9 | 3 | 5 | 8 | 2 | 6 | 7 | 3 | 5 | 8 | 1 | 4 | 9 | 2 | 6 | 7 |
| sub39 | active | 4 | 9 | 3 | 5 | 8 | 2 | 6 | 7 | 1 | 5 | 8 | 1 | 4 | 9 | 2 | 6 | 7 | 3 |
| sub40 | sham | 4 | 9 | 3 | 5 | 8 | 2 | 6 | 7 | 1 | 5 | 8 | 1 | 4 | 9 | 2 | 6 | 7 | 3 |
| sub41 | active | 9 | 3 | 5 | 8 | 2 | 6 | 7 | 1 | 4 | 8 | 1 | 4 | 9 | 2 | 6 | 7 | 3 | 5 |
| sub42 | sham | 9 | 3 | 5 | 8 | 2 | 6 | 7 | 1 | 4 | 8 | 1 | 4 | 9 | 2 | 6 | 7 | 3 | 5 |
| sub44 | sham | 3 | 5 | 8 | 2 | 6 | 7 | 1 | 4 | 9 | 1 | 4 | 9 | 2 | 6 | 7 | 3 | 5 | 8 |
| sub45 | active | 5 | 8 | 2 | 6 | 7 | 1 | 4 | 9 | 3 | 4 | 9 | 2 | 6 | 7 | 3 | 5 | 8 | 1 |
| sub46 | sham | 5 | 8 | 2 | 6 | 7 | 1 | 4 | 9 | 3 | 4 | 9 | 2 | 6 | 7 | 3 | 5 | 8 | 1 |
| sub47 | active | 8 | 2 | 6 | 7 | 1 | 4 | 9 | 3 | 5 | 9 | 2 | 6 | 7 | 3 | 5 | 8 | 1 | 4 |
| sub48 | sham | 8 | 2 | 6 | 7 | 1 | 4 | 9 | 3 | 5 | 9 | 2 | 6 | 7 | 3 | 5 | 8 | 1 | 4 |
| sub49 | active | 2 | 6 | 7 | 1 | 4 | 9 | 3 | 5 | 8 | 2 | 6 | 7 | 3 | 5 | 8 | 1 | 4 | 9 |
| sub50 | sham | 2 | 6 | 7 | 1 | 4 | 9 | 3 | 5 | 8 | 2 | 6 | 7 | 3 | 5 | 8 | 1 | 4 | 9 |
| sub51 | active | 6 | 7 | 1 | 4 | 9 | 3 | 5 | 8 | 2 | 6 | 7 | 3 | 5 | 8 | 1 | 4 | 9 | 2 |
| sub52 | sham | 6 | 7 | 1 | 4 | 9 | 3 | 5 | 8 | 2 | 6 | 7 | 3 | 5 | 8 | 1 | 4 | 9 | 2 |
| sub53 | active | 7 | 1 | 4 | 9 | 3 | 5 | 8 | 2 | 6 | 7 | 3 | 5 | 8 | 1 | 4 | 9 | 2 | 6 |
| sub54 | sham | 7 | 1 | 4 | 9 | 3 | 5 | 8 | 2 | 6 | 7 | 3 | 5 | 8 | 1 | 4 | 9 | 2 | 6 |
| sub55 | active | 7 | 6 | 2 | 8 | 5 | 3 | 9 | 4 | 1 | 8 | 5 | 3 | 7 | 6 | 2 | 9 | 4 | 1 |

**Appendix 4 The Montreal Neurological Institute coordinates and the mapping Brodmann area with Percentage of overlap of each channel**

|  |  | **MNI coordinate position** | | |  |  |
| --- | --- | --- | --- | --- | --- | --- |
| **channel** | **S-D** | **x** | **y** | **z** | **Brodmann** | **Percentage of Overlap** |
| **CH01** | **S1-D2** | 73 | -23 | 8 | 22 - Superior Temporal Gyrus | 0.76 |
| **CH02** | **S2-D1** | 60 | -67 | 12 | 37 - Fusiform gyrus | 0.67 |
| **CH03** | **S2-D2** | 68 | -49 | 23 | 22 - Superior Temporal Gyrus | 0.74 |
| **CH04** | **S2-D3** | 53 | -74 | 29 | 39 - Angular gyrus | 0.98 |
| **CH05** | **S3-D2** | 70 | -25 | 30 | 2 - Primary Somatosensory Cortex | 0.82 |
| **CH06** | **S4-D4** | 64 | 13 | 24 | 6 - Pre-Motor and Supplementary Motor Cortex | 0.57 |
| **CH07** | **S4-D5** | 55 | 20 | -9 | 38 - Temporopolar area | 1.00 |
| **CH08** | **S4-D6** | 59 | 32 | 15 | 45 - pars triangularis Broca's area | 1.00 |
| **CH09** | **S5-D4** | 53 | 17 | 44 | 44 - pars opercularis, part of Broca's area | 0.46 |
| **CH10** | **S5-D6** | 49 | 35 | 35 | 45 - pars triangularis Broca's area | 0.64 |
| **CH11** | **S5-D7** | 35 | 34 | 51 | 9 - Dorsolateral prefrontal cortex | 0.77 |
| **CH12** | **S6-D5** | 52 | 41 | -15 | 47 - Inferior frontal gyrus | 0.74 |
| **CH13** | **S6-D6** | 51 | 50 | 4 | 46 - Dorsolateral prefrontal cortex | 0.82 |
| **CH14** | **S6-D8** | 41 | 62 | -9 | 47 – Inferior frontal gyrus | 0.35 |
| **CH15** | **S7-D6** | 41 | 53 | 26 | 46 - Dorsolateral prefrontal cortex | 0.83 |
| **CH16** | **S7-D7** | 26 | 51 | 43 | 9 - Dorsolateral prefrontal cortex | 0.94 |
| **CH17** | **S7-D8** | 31 | 67 | 12 | 10 - Frontopolar area | 0.95 |
| **CH18** | **S7-D9** | 16 | 64 | 32 | 10 - Frontopolar area | 0.62 |
| **CH19** | **S8-D7** | 12 | 44 | 55 | 9 - Dorsolateral prefrontal cortex | 0.54 |
| **CH20** | **S8-D9** | 0 | 53 | 43 | 9 - Dorsolateral prefrontal cortex | 1.00 |
| **CH21** | **S8-D10** | -11 | 44 | 54 | 9 - Dorsolateral prefrontal cortex | 0.60 |
| **CH22** | **S9-D8** | 16 | 72 | -8 | 11 - Orbitofrontal area | 0.78 |
| **CH23** | **S9-D9** | 2 | 68 | 13 | 10 - Frontopolar area | 1.00 |
| **CH24** | **S9-D11** | -13 | 72 | -7 | 11 - Orbitofrontal area | 0.70 |
| **CH25** | **S10-D9** | -13 | 63 | 33 | 10 - Frontopolar area | 0.56 |
| **CH26** | **S10-D10** | -22 | 51 | 43 | 9 - Dorsolateral prefrontal cortex | 0.96 |
| **CH27** | **S10-D11** | -27 | 67 | 14 | 10 - Frontopolar area | 0.93 |
| **CH28** | **S10-D12** | -39 | 53 | 27 | 46 - Dorsolateral prefrontal cortex | 0.86 |
| **CH29** | **S11-D10** | -31 | 33 | 51 | 9 - Dorsolateral prefrontal cortex | 0.70 |
| **CH30** | **S11-D12** | -46 | 35 | 35 | 45 - pars triangularis Broca's area | 0.62 |
| **CH31** | **S11-D14** | -50 | 18 | 45 | 9 - Dorsolateral prefrontal cortex | 0.49 |
| **CH32** | **S12-D11** | -39 | 61 | -8 | 10 - Frontopolar area | 0.33 |
| **CH33** | **S12-D12** | -50 | 48 | 4 | 46 - Dorsolateral prefrontal cortex | 0.69 |
| **CH34** | **S12-D15** | -50 | 40 | -15 | 47 - Inferior frontal gyrus | 0.69 |
| **CH35** | **S13-D12** | -56 | 31 | 15 | 45 - pars triangularis Broca's area | 0.99 |
| **CH36** | **S13-D14** | -61 | 12 | 25 | 6 - Pre-Motor and Supplementary Motor Cortex | 0.51 |
| **CH37** | **S13-D15** | -54 | 17 | -9 | 38 - Temporopolar area | 0.96 |
| **CH38** | **S14-D13** | -58 | -68 | 26 | 39 - Angular gyrus | 0.93 |
| **CH39** | **S14-D16** | -63 | -63 | 9 | 37 - Fusiform gyrus | 0.90 |
| **CH40** | **S14-D17** | -68 | -45 | 20 | 22 - Superior Temporal Gyrus | 0.84 |
| **CH41** | **S15-D17** | -68 | -21 | 29 | 2 - Primary Somatosensory Cortex | 0.65 |
| **CH42** | **S16-D17** | -69 | -20 | 9 | 22 - Superior Temporal Gyrus | 0.77 |

**Appendix 5 Centrality measures per node in the high-load effort condition**

| **Centrality measures per variable** | | | | | | | | | |
| --- | --- | --- | --- | --- | --- | --- | --- | --- | --- |
|  | | **Network** | | | | | | | |
| **Variable** | | **Betweenness** | | **Closeness** | | **Strength** | | **Expected influence** | |
| ch1 |  | -0.751 |  | 0.000 |  | -0.452 |  | -0.452 |  |
| ch6 |  | 0.300 |  | 0.000 |  | 1.137 |  | 1.137 |  |
| ch7 |  | -0.751 |  | 0.000 |  | -1.162 |  | -1.162 |  |
| ch34 |  | 1.352 |  | 0.000 |  | 1.355 |  | 1.355 |  |
| ch37 |  | -0.751 |  | 0.000 |  | 0.264 |  | 0.264 |  |
| win |  | -0.751 |  | 0.000 |  | -1.116 |  | -1.116 |  |
| miss |  | 1.352 |  | 0.000 |  | -0.026 |  | -0.026 |  |


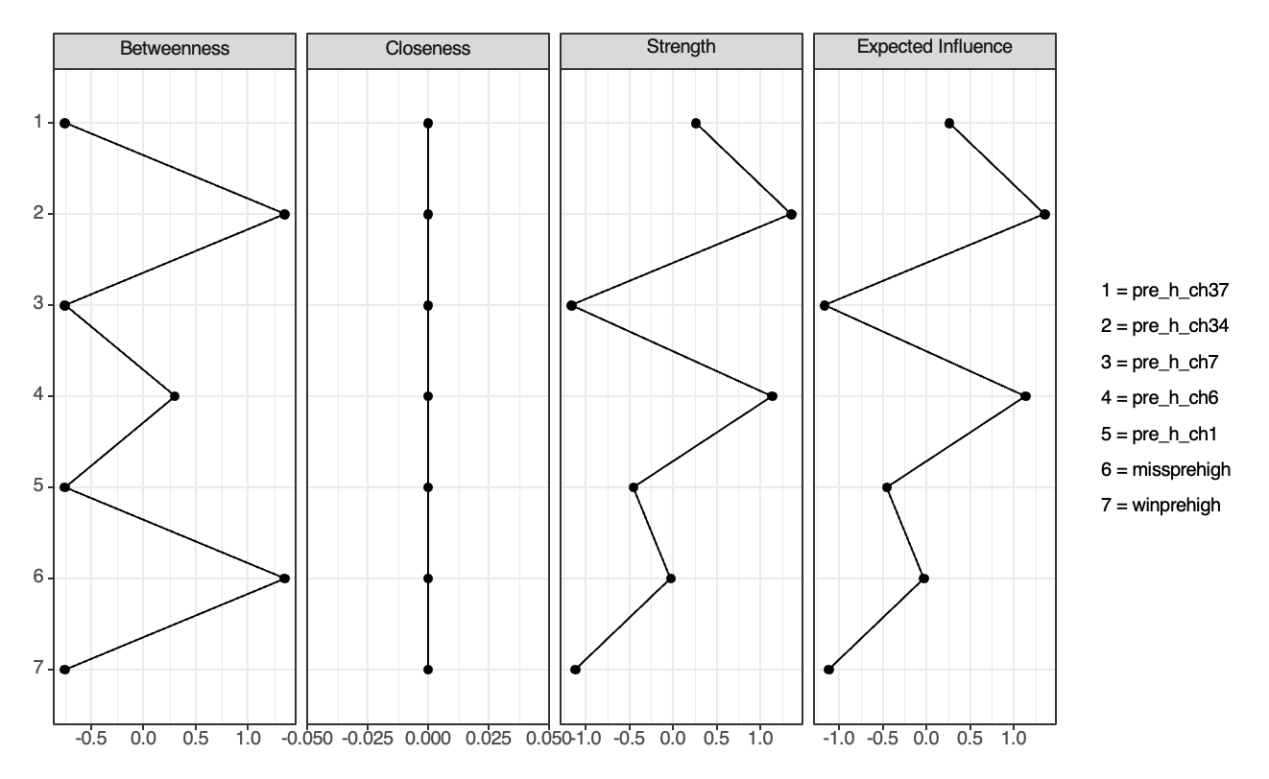

Supplement: Supplementary Materials — Appendix S1: sample size calculation. Appendix S2: manipulation test of effort conditions. Appendix S3: the pseudo-random order matrix of presented nine blocks. Appendix S4: the Montreal Neurological Institute coordinates and the mapping Brodmann area with percentage of overlap of each channel. Appendix S5: centrality measures per node in the high-load effort condition. [file 3135532.f1.docx]
